# Oxidative Stress and Cancer Therapy: Controlling Cancer Cells Using Reactive Oxygen Species

**DOI:** 10.3390/ijms252212387

**Published:** 2024-11-18

**Authors:** Songhyun Ju, Manish Kumar Singh, Sunhee Han, Jyotsna Ranbhise, Joohun Ha, Wonchae Choe, Kyung-Sik Yoon, Seung Geun Yeo, Sung Soo Kim, Insug Kang

**Affiliations:** 1 Department of Biochemistry and Molecular Biology, School of Medicine, Kyung Hee University, Seoul 02447, Republic of Korea; thdgus8543@khu.ac.kr (S.J.); manishbiochem@gmail.com (M.K.S.); sunheehan@khu.ac.kr (S.H.); jogm25@khu.ac.kr (J.R.); hajh@khu.ac.kr (J.H.); wchoe@khu.ac.kr (W.C.); sky9999@khu.ac.kr (K.-S.Y.); 2 Biomedical Science Institute, Kyung Hee University, Seoul 02447, Republic of Korea; 3 Department of Biomedical Science, Graduate School, Kyung Hee University, Seoul 02447, Republic of Korea; 4 Department of Otorhinolaryngology—Head and Neck Surgery, College of Medicine, Kyung Hee University Medical Center, Kyung Hee University, Seoul 02453, Republic of Korea; yeo2park@gmail.com

**Keywords:** cancer, reactive oxygen species (ROS), oxidative stress

## Abstract

Cancer is a multifaceted disease influenced by various mechanisms, including the generation of reactive oxygen species (ROS), which have a paradoxical role in both promoting cancer progression and serving as targets for therapeutic interventions. At low concentrations, ROS serve as signaling agents that enhance cancer cell proliferation, migration, and resistance to drugs. However, at elevated levels, ROS induce oxidative stress, causing damage to biomolecules and leading to cell death. Cancer cells have developed mechanisms to manage ROS levels, including activating pathways such as NRF2, NF-κB, and PI3K/Akt. This review explores the relationship between ROS and cancer, focusing on cell death mechanisms like apoptosis, ferroptosis, and autophagy, highlighting the potential therapeutic strategies that exploit ROS to target cancer cells.

## 1. Introduction

Cancer is a multifaceted disease marked by the uncontrolled proliferation and division of cells within specific tissues of the body. Globally, it poses significant health challenges, particularly when not diagnosed at an early stage, leading to high mortality rates [1]. Various factors contribute to cancer proliferation and progression; among these, ROS play a dual role, acting both in the initiation and progression of cancer as well as in its suppression and treatment. Many research groups have demonstrated the association of ROS with multiple cancer types, including breast, colon, lung, hepatocellular, and cervical cancers [2]. Oxidative stress, primarily driven by ROS, plays a significant role in cancer development and progression and contributes to other pathological conditions, such as diabetes, metabolic disorders, and atherosclerosis, which amplify these harmful effects [3].

**Figure 1:**
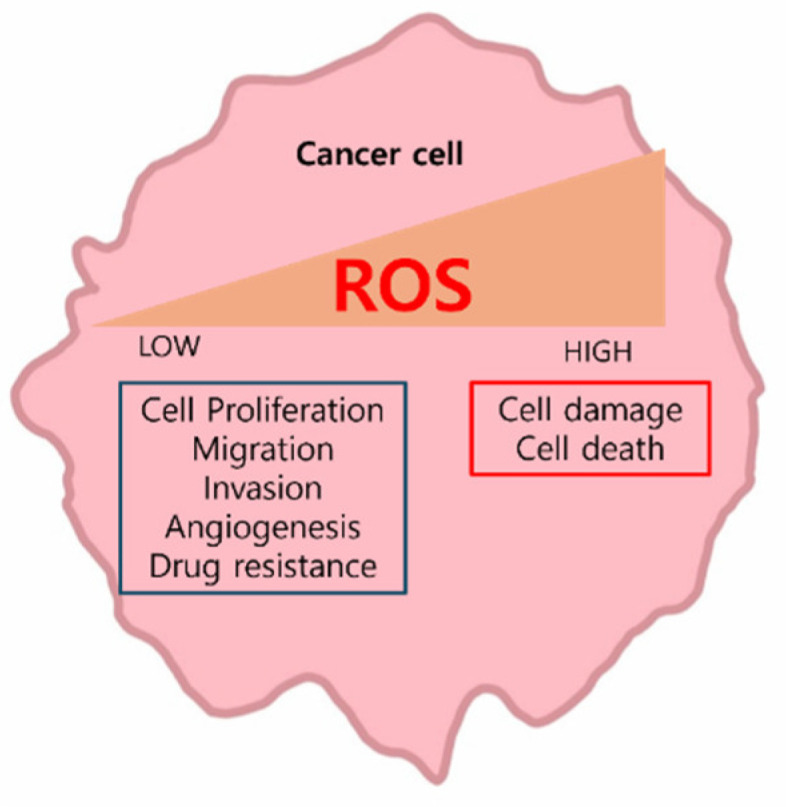
Functions dependent on ROS levels in cancer cells.

ROS are derived from various reactive oxygen species generated during metabolic processes, including mitochondrial oxidative phosphorylation (OXPHOS) [4]. They are crucial to numerous biological processes [4]. ROS function in a dual capacity: they act as signaling molecules that activate proliferation, migration, invasion, angiogenesis, and drug resistance pathways in cancer cells. Conversely, elevated ROS levels cause oxidative damage to proteins, nucleic acids, lipids, cell membranes, and organelles, ultimately leading to cell death (Figure 1) [5]. In this review, we provide a comprehensive analysis of how cancer cells utilize the ROS pathway and discuss strategies for leveraging ROS for therapeutic intervention in cancer.

## 2. Cancer

Cancer cells favor glycolysis over oxidative phosphorylation, even when oxygen levels are normal [6]. This metabolic reprogramming is accompanied by the upregulation of glucose transporters, which compensates for glycolysis’s approximately 18-fold lower energy efficiency compared to OXPHOS [7]. However, it is inaccurate to generalize that all cancer cells rely solely on glycolysis in place of OXPHOS. Instead, the preference for glycolysis is modulated by a range of factors, including oncogene activation, the loss of tumor suppressor function, the hypoxic tumor microenvironment, mutations in mitochondrial DNA (mtDNA), and the genetic background of the cancer [8]. Cancer cells also frequently utilize the glutamine metabolism to fuel the tricarboxylic acid (TCA) cycle, which is critical for maintaining cellular energy, biosynthesis, and proliferation [9].

Furthermore, the upregulation of the pentose phosphate pathway (PPP) and enhanced mitochondrial biosynthesis are observed in many cancer cells, helping to support ROS detoxification and maintain redox homeostasis within the cell [10,11,12].

## 3. ROS

### 3.1. ROS Generation, Types, and Regulation

Free radicals are molecules that contain unpaired electrons, which endows them with notable reactivity [13]. ROS are highly reactive molecules formed from oxygen (O_2_), water, and hydrogen peroxide (H_2_O_2_). They actively participate in various chemical reactions and biological processes [14]. ROS are generally classified into two main categories: free radical species, which include the hydroxyl radical (•OH) and superoxide anion (O_2_•−), and non-radical molecules, such as H_2_O_2_ [15]. Among these, the hydroxyl radical is the most aggressive oxidant, typically generated through reactions involving superoxide anion and hydrogen peroxide [16]. ROS are produced as byproducts of several biochemical reactions occurring in cellular organelles like mitochondria, peroxisomes, the cytochrome P-450 system, NADPH oxidase, and other cellular components [17]. In addition to ROS, reactive nitrogen species (RNS) are formed during nitric oxide synthase (NOS)-catalyzed reactions, where L-arginine is converted to L-citrulline, producing nitric oxide (NO) as a byproduct. Although NO itself is not highly reactive, it can interact with other molecules, such as O_2_ and O_2_•−, as well as transition metals. These interactions lead to the formation of reactive intermediates like peroxynitrite (ONOO−), which are highly reactive and can inactivate proteins and damage other macromolecules (Table 1) [18]. ROS can also be induced by various external factors. For instance, cigarette smoke contains several oxidants, including free radicals, superoxide, and nitrogen monoxide, all contributing to ROS production. Exposure to ozone initiates lipid peroxidation and releases inflammatory mediators, thereby increasing oxidative stress [19].

**Figure 2:**
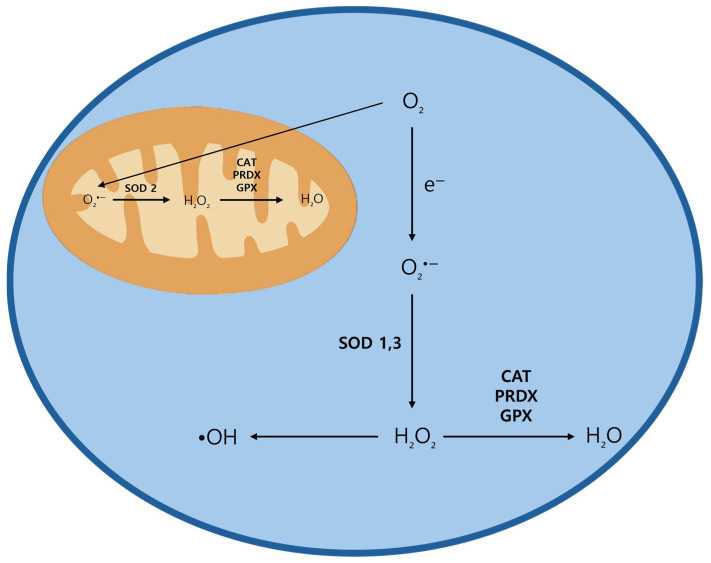
Antioxidant enzymes and their mechanisms.

At low to moderate concentrations, ROS facilitate essential cellular functions such as proliferation, migration, and invasion. However, at higher levels, ROS inflict damage upon biomolecules, leading to cellular injury and cell death [5]. To regulate ROS, cells utilize several antioxidant enzymes, including superoxide dismutase (SOD), catalase (CAT), peroxiredoxin (PRDX), and glutathione peroxidase (GPX). These enzymes work in concert to neutralize highly reactive species like O_2_•− and other ROS, transforming them into less reactive molecules, such as H_2_O_2_, which is subsequently converted into water (Figure 2) [20]. ROS also activate multiple signaling pathways, notably the NRF2/KEAP1 pathway (nuclear factor erythroid 2-related factor 2/Kelch-like ECH-associated protein 1), a critical mechanism for regulating ROS levels. When ROS modify KEAP1, they inhibit its ability to degrade NRF2, allowing NRF2 to translocate into the nucleus and promote the expression of cytoprotective genes. Additionally, other pathways such as nuclear factor-κB (NF-κB), phosphoinositide 3-kinase (PI3K)/AKT, and mitogen-activated protein kinase (MAPK) are activated. These pathways play key roles in inflammatory and immune responses and contribute to cell growth, survival, or apoptosis, depending on cellular context [21].

**Table 1:** Role in cancer by ROS type.

ROS	Role in Cancer	References
Superoxide (O_2−_)	Promotes tumor growth and metastasis by enhancing cell proliferation and migration. Can induce cell death at high levels.	[22,23]
Hydroxyl radical (•OH)	Causes severe DNA damage and mutations, promoting cancer progression. Excessive levels can trigger apoptosis, killing cancer cells.	[24,25,26]
Hydrogen peroxide (H_2_O_2_)	It functions as a signaling molecule that supports the survival of cancer cells. However, at elevated concentrations, it causes oxidative stress, resulting in cell death.	[26,27]
Peroxynitrite (ONOO^−^)	Facilitates tumor angiogenesis and inflammation. However, excessive oxidative stress can damage cancer cells and suppress tumor growth.	[28,29]
Nitric oxide (NO)	Low levels promote cancer growth, while high levels trigger apoptosis and inhibit tumor development.	[30]

### 3.2. Interaction Between ROS and Macromolecules

As a result of oxidative stress, macromolecules such as DNA, RNA, proteins, and lipids are major targets of ROS [31]. Oxidative stress develops when the body’s antioxidant mechanisms are outstripped by the excessive generation of free radicals, thereby failing to neutralize these reactive entities [32]. ROS can modify or degrade lipids with hydroxyl groups in their polar components, such as phospholipids, ceramides, diacylglycerols, and acylamides [33]. The cell membrane is composed of a substantial number of polyunsaturated fatty acids (PUFAs), rendering it particularly susceptible to damage caused by ROS. This results in the peroxidation of cell membrane lipids, which affects the fluidity and deformability of the cell membrane [34]. Lipid peroxidation triggers a chain reaction that generates toxic byproducts, including lipid hydroperoxides and aldehydes, such as malondialdehyde, propionaldehyde, hexanal, and 4-hydroxynonenal, all of which are cytotoxic and mutagenic [35,36]. Protein oxidation due to ROS plays a significant role in aging and disease development [18]. ROS can oxidize amino acid residues, break peptide bonds, and cause protein aggregation [37]. In DNA, ROS-induced damage includes single- and double-strand breaks, nucleotide base modifications, deoxyribose sugar alterations, DNA cross-linking, and impaired DNA-binding abilities, all of which can lead to carcinogenesis [2,38]. Specifically, ROS oxidize guanine to produce 8-oxo-G, a mutagenic and carcinogenic lesion [38]. During DNA replication, 8-oxo-G can erroneously pair with adenine as well as cytosine, leading to G→T mutations commonly observed in oxidative stress-related cancers, including those of the lung, breast, ovary, stomach, and colon [39]. Approximately 90% of these oxidized bases are repaired via single nucleotide excision repair, with the remaining 10% corrected through long-patch base excision repair pathways [40]. mtDNA is more vulnerable to oxidative damage than nuclear DNA due to the lack of protective histones and the absence of certain DNA repair pathways, such as single nucleotide excision repair, that are present in the nucleus. This makes mtDNA particularly susceptible to ROS, establishing a connection between oxidative damage to mitochondrial DNA and carcinogenesis [40]. In prostate and primary breast cancer (the original tumor in breast tissue prior to metastasis), oxidative damage to mtDNA contributes to changes in gene expression and somatic mutations, linking mitochondrial dysfunction to cancer progression [41,42,43].

### 3.3. Signaling Associated with ROS

#### 3.3.1. NRF2

NRF2 was initially regarded as a tumor suppressor gene, as early studies indicated that NRF2 deficiency increased cancer susceptibility in various types, such as colon cancer and melanoma [44,45,46]. However, recent findings suggest that NRF2 may also act as a tumor promoter in specific cancers, particularly lung adenocarcinoma (LUAD) and lung squamous cell carcinoma (LUSC) [47]. The activation of NRF2 triggers the transcription of numerous genes involved in the antioxidant response, offering cellular protection against oxidative stress [48]. As a redox-sensitive transcription factor, NRF2 plays critical roles in anti-apoptotic signaling, cell cycle regulation, cell proliferation, and protein homeostasis through mechanisms such as autophagy and proteasomal degradation. NRF2 also indirectly influences heme and iron metabolism and xenobiotic transport by regulating the expression of genes in these pathways, enhancing cellular resilience against oxidative stress and various other stressors [44]. This protective mechanism includes the upregulation of enzymes such as glutathione S-transferases and heme oxygenase-1, which detoxify ROS and mitigate cellular damage [49]. As such, NRF2’s protective functions may inadvertently support cancer cell survival, growth, transformation, metastasis, and chemotherapy resistance [50]. Studies further indicate that patients with high NRF2 levels in tumor tissues tend to have an increased risk of recurrence and a poorer prognosis [51]. Despite numerous studies and reviews on NRF2’s dual role in cancer biology, the development of effective NRF2 inhibitors remains a significant challenge in the field [50].

#### 3.3.2. NF-κB

The transcription factor NF-κB is activated in various tumor types, where it functions as an essential regulator of genes involved in immune responses, cell growth, apoptosis, and inflammation [52]. NF-κB typically resides in the cytoplasm in an inactive form, but upon activation, it translocates to the nucleus, initiating the transcription of genes essential for these processes [52]. ROS can either activate or inhibit NF-κB signaling, and when activated by ROS, NF-κB helps to manage oxidative stress by reducing ROS accumulation, thereby promoting cell survival through anti-apoptotic mechanisms [53].

NF-κB has a multifaceted role, particularly in immune regulation and as an anti-apoptotic survival factor that enables immune cells to resist cell death during infections [54]. In acute inflammatory responses, NF-κB activation typically resolves without significant complications. However, in chronic inflammation, it can allow precancerous cells to evade immune detection and may support tumorigenesis [55]. In numerous cancers, NF-κB is constitutively active, generating signals that enhance cell survival and inhibit apoptosis [56,57]. The hyperactivation of the NF-κB pathway promotes cell proliferation by inducing the transcription of the cyclin D1 gene, critical for cell cycle progression from the G₁ to S phase [58]. Additionally, the NF-κB-driven transcription of inflammatory cytokines may act as growth factors for tumor cells, supporting angiogenesis and contributing to metastasis [59,60]. Furthermore, persistent NF-κB activation induces telomerase reverse transcriptase (TERT) activity, which protects telomeres from shortening and grants cells extended replication potential, thus supporting tumor survival [61].

NF-κB activity is also modulated by glycogen synthase kinase-3 beta (GSK3β), which phosphorylates NF-κB essential modifier (NEMO), a key component for NF-κB activity [62]. The activation of GSK3β enhances NF-κB-driven transcription of inflammation and metastasis-related genes that are dysregulated in cancer [63]. A range of NF-κB inhibitors is currently in clinical use and demonstrate potential as anticancer agents [52]. These inhibitors include compounds that disrupt IKK activity, agents that bind to NF-κB to block nuclear translocation, and proteasome inhibitors that prevent NF-κB activation by halting the degradation of inhibitory proteins such as IκB [64,65,66,67,68].

#### 3.3.3. PI3K/AKT

The PI3K/Akt signaling pathway is critical for multiple cellular functions, yet it frequently becomes dysregulated in cancer, promoting tumor growth and progression [69]. This pathway begins when phosphatidylinositol-4,5-bisphosphate (PIP_2_) is phosphorylated by PI3K to produce phosphatidylinositol-3,4,5-trisphosphate (PIP_3_), which activates various oncogenic kinases, including Akt, a serine/threonine kinase. Once activated, Akt recruits several downstream signaling proteins involved in cell survival and growth [70].

ROS levels also influence PI3K/Akt signaling, as excessive ROS inhibit this pathway, leading to cell death and inflammation, whereas low to moderate ROS levels activate PI3K/Akt signaling to inhibit apoptosis and stimulate cell proliferation [71]. Upon activation, Akt phosphorylates multiple substrates that enhance cancer progression, particularly through the mechanistic target of rapamycin (mTOR). In cancer cells, the PI3K/Akt/mTOR pathway is often aberrantly activated through various mutations [72,73]. For instance, activating mutations in the PIK3CA gene have been identified in a wide range of cancers, including breast, endometrial, cervical, colorectal, esophageal, gallbladder, non-small cell lung, ovarian, and gastric cancers [74]. The AKT1 E17K mutation is notably frequent in breast cancer, while AKT2 amplification or overexpression is common in breast, ovarian, and prostate cancers. Similarly, AKT3 amplification has been observed in cancers such as breast, endometrial, melanoma, ovarian epithelial tumors, cholangiocarcinoma, and non-small cell lung cancer [75,76,77,78]. The overexpression of mTOR has been reported in ovarian, urothelial, and skin cancers, while downregulation is noted in central nervous system tumors. Additionally, mTOR mutations are seen in meningeal, endometrial, and endometrioid cancers [79]. Approximately 70% of ovarian or breast cancers and up to 90% of LUAD show an abnormal activation of the PI3K/Akt/mTOR pathway [79]. mTOR is a central regulator of biological processes such as tumor growth, cell survival, metabolism, and immunity. It exists in complexes, such as mTORC1 and mTORC2, which each play essential roles in these functions [80]. A related genetic disorder, tuberous sclerosis complex (TSC), is characterized by hamartomas in various organs and results from mutations in the TSC1 and TSC2 tumor suppressor genes. The TSC1–TSC2 complex acts to inhibit p70 S6K1 (p70 ribosomal protein S6 kinase 1), thereby reducing mTORC1 activity, protein synthesis, and cell growth. Additionally, the TSC1–TSC2 complex activates 4E-BP1 (eukaryotic initiation factor 4E binding protein 1), further diminishing protein synthesis and restricting cell growth [81].

GSK3, a significant downstream target, has two isoforms: GSK3α and GSK3β, both involved in the insulin-regulated process of glycogen synthesis [82]. GSK3 is active in numerous biochemical processes and disease states [83]. It can function as either a tumor suppressor or a promoter of cell proliferation, depending on cellular context [84]. Specifically, GSK3β overexpression has been shown to induce the production of the anti-apoptotic protein BCL-XL, contributing to resistance against apoptosis initiated by tumor necrosis factor-related apoptosis-inducing ligand (TRAIL) [85]. Elevated GSK3β levels are observed in several cancers, including ovarian, colorectal, and pancreatic cancers [86]. Since GSK3β is a known negative regulator of NRF2, its activity may increase ROS accumulation, thereby promoting oxidative stress [87]. In advanced papillomas and squamous cell carcinomas, phosphorylated GSK3β(Ser9)—an inactive form of GSK3β—is markedly elevated, while the active form, phosphorylated GSK3β(Tyr216), is significantly decreased in squamous cell carcinoma tissues compared to normal tissues [88]. Thus, cancer treatments targeting the activation of GSK3β could induce ROS accumulation, potentially leading to cancer cell death.

#### 3.3.4. MAPK

The RAS/RAF/MEK/ERK (MAPK) signaling pathway can be activated by ROS [89]. ROS promote MAPK pathway activation by oxidizing and modifying key regulatory proteins, including MAP kinase kinase kinases (MAPKKKs). For instance, ROS can oxidize Apoptotic Signal-Regulating Kinase 1 (ASK1), resulting in its activation. Once activated, ASK1 phosphorylates MAP kinase kinases (MAPKKs) such as MKK4/7, which subsequently activate downstream MAP kinases [90].

The MAPK pathway is one of the most frequently dysregulated signaling pathways in human cancers, with mutations commonly affecting RAS and RAF genes [91]. Approximately 30% of all human tumors harbor mutations in one of the canonical RAS genes, particularly in cancers such as pancreatic ductal adenocarcinoma, colorectal cancer, non-small cell lung cancer, malignant melanoma, bladder cancer, thyroid cancer, and certain hematopoietic malignancies [92]. RAF mutations are prevalent in cancers including malignant melanoma, papillary thyroid cancer, colorectal cancer, and ovarian cancer [93]. As an integral component of cellular signaling, the RAS protein functions as a regulatory switch, binding to either guanosine triphosphate (GTP) or guanosine diphosphate (GDP). Upon activation, RAS binds GTP and subsequently activates RAF, which in turn phosphorylates MEK1 and MEK2 proteins. These MEK proteins then activate ERK1 and ERK2, propagating the MAPK signaling cascade [94]. This pathway plays a pivotal role in the pathogenesis of cancer by influencing cell proliferation, differentiation, migration, survival, and death [95,96]. Moreover, MAPKs such as JNK are instrumental in regulating transcription, cell proliferation, apoptosis, inflammation, metastasis, and angiogenesis—all processes that are critical for tumor progression [97]. Conversely, the MAPK pathway, particularly through the p38 MAPK axis, also supports tumor suppression by inhibiting cell proliferation, promoting oncogene-induced senescence, and initiating DNA damage responses, all of which contribute to an inflammatory response that can hinder tumor development [98]. Depending on the cellular context and type, MAPK pathways can have diverse effects, alternately contributing to cancer progression or suppression [97].

Elevated MAPK pathway activity is significantly associated with the progression of neoplastic growth [99]. Numerous pharmaceutical agents targeting the MAPK pathway have been developed for the treatment of cancers [100]. Thus, strategies that simultaneously inhibit MAPK activation and harness ROS-induced cell death offer promising avenues for the development of effective ROS-based cancer therapies.

## 4. Cell Death Pathways Related to ROS

### 4.1. Ferroptosis

**Figure 3:**
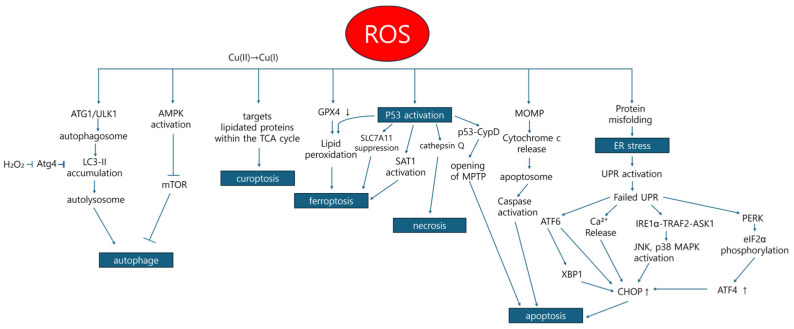
Cell death associated with ROS.

Ferroptosis is a distinct form of cell death separate from apoptosis, relying on both lipids and ROS (Figure 3) [101]. It is characterized by the oxidation of membrane phospholipids containing PUFAs, the Fenton reaction, and an impaired capacity to repair lipid hydroperoxides [34]. Cancer cells, which are often highly iron-dependent, are especially susceptible to ferroptosis compared to noncancerous cells [102]. Additionally, patients with hemochromatosis—a metabolic disorder causing excessive iron absorption and storage—have an increased risk of developing several cancers, including liver, colon, rectal, prostate, and breast cancer [103].

Under typical conditions, lipid peroxides are reduced to lipid alcohols (e.g., PE-OH) by reductase enzymes, which protects cells from oxidative stress. However, when an excess of lipid peroxides (e.g., PE-OOH) accumulates, this protective mechanism becomes ineffective [104]. The reduction in lipid peroxides to lipid alcohols is primarily catalyzed by GPX4 and its cofactor, glutathione (GSH) [105]. In the absence of functional GPX4, or when GPX4 activity is compromised, lipid peroxides build up, resulting in ferroptotic cell death [106]. Research has shown that GPX4 expression is significantly elevated in a variety of tumor types, including colorectal, renal choriocarcinoma, renal clear cell carcinoma, lung, lung adenocarcinoma, prostate, rectal, thyroid, and endometrial cancers [107,108].

Several small-molecule compounds, such as erastin, RSL3, FIN56, and FINO2, have been identified as GPX4 inhibitors [109,110,111,112,113]. Due to erastin’s low water solubility and metabolic instability, modified versions like piperazine erastin (PE) and imidazole ketone erastin (IKE) have been developed. Both PE and IKE have demonstrated tumor-suppressing effects in B-cell lymphoma xenograft models [114]. RSL3 has also been shown to induce ferroptosis, and its anticancer potential, particularly in iron-rich tissues like the liver, is actively under investigation [115].

### 4.2. ER Stress

The endoplasmic reticulum (ER) is crucial for various cellular processes, including protein folding, processing, and transport; lipid and steroid synthesis; calcium storage and regulation; and cellular detoxification [116]. The accumulation of ROS in cancer cells can lead to ER stress, ultimately resulting in apoptosis. ROS have been shown to promote the release of Ca^2^⁺ from the ER lumen, with Ca^2^⁺ acting as a signaling molecule to regulate chaperone proteins involved in protein folding. Consequently, ER stress triggered by ROS arises due to the buildup of misfolded proteins within the ER lumen (Figure 3) [117].

In response to ER stress, cells activate a set of signaling pathways collectively known as the unfolded protein response (UPR). In mammals, UPR is detected by three primary sensors: inositol-requiring enzyme 1 alpha (IRE1α), activating transcription factor 6 (ATF6), and protein kinase RNA-like ER kinase (PERK) [118]. PERK, IRE1α, and ATF6 function to increase the expression of ER chaperones, temporarily halt mRNA translation, and limit protein entry into the ER, thereby mitigating the accumulation of misfolded proteins [119].

However, when UPR fails to relieve ER stress, it can lead to cell death. Specifically, IRE1α recruits TRAF2 and ASK1 to activate JNK and p38 MAPK, which subsequently phosphorylate and activate CHOP, contributing to apoptosis. Similarly, PERK phosphorylates eIF2α, enhancing ATF4 translation and promoting CHOP expression, a pro-apoptotic gene [120]. Additionally, decreased cytosolic calcium within the ER can lead to mitochondrial calcium overload, triggering apoptosis via the Bcl-2-dependent pathway [121]. Furthermore, ATF6 directly activates both CHOP and XBP1; activated XBP1 can, in turn, further promote CHOP expression, intensifying the pro-apoptotic response [122].

There is an increasing interest in studying natural compounds that target ER stress-mediated cell death for potential cancer therapies, particularly in cancers of the lung, breast, colon, and stomach [123]. For instance, compounds from *Oryza officinalis*, *Curcuma longa*, and *Mylabris phalerata Pallas* have been shown to induce ER stress through elevated ROS levels. For example, Ω-Hydroxyundec-9-enoic acid (Ω-HUA) from *Oryza officinalis* activates ER stress responses, increases ROS, and upregulates apoptotic proteins. When ROS scavengers were applied, Ω-HUA-induced cell death and ER stress were prevented. Similarly, curcumin treatment activated ER stress and apoptotic proteins, and ROS scavenging also reversed ER stress and cell death [124,125,126]. Thus, leveraging these natural compounds to induce ER stress and cell death through ROS generation could offer a promising therapeutic approach for cancer treatment.

### 4.3. Apoptosis

Mitochondria serve as both a source and target of ROS. Elevated levels of ROS within mitochondria trigger apoptosis, leading to mitochondrial cell death through the intrinsic pathway. During apoptosis initiation, cytochrome c is released from the intermembrane space of mitochondria into the cytoplasm (Figure 3) [127]. In its usual state, cytochrome c resides in the intracrystalline space of the inner mitochondrial membrane, where it functions in the mitochondrial electron transport chain [128].

The increased permeability of the outer mitochondrial membrane (OMM) facilitates the release of several pro-apoptotic proteins into the cytoplasm, including cytochrome c, apoptosis-inducing factor (AIF), endonuclease G (endoG), and Smac/Diablo (a mitochondrial-derived caspase activator and apoptosis inhibitor at low pH) [129]. Once in the cytoplasm, cytochrome c associates with Apaf-1 and caspase 9 to form a complex known as the “apoptosome”, which subsequently activates caspase-3 [130]. The activation of caspase-3 cleaves the inhibitor of caspase-activated deoxyribonuclease, initiating caspase-activated deoxyribonuclease activity and ultimately leading to nuclear apoptosis [131].

AIF and EndoG translocate to the nucleus, where they participate in caspase-independent apoptosis. While the exact role of EndoG is still under investigation, evidence suggests that AIF interacts with DNA, causing chromatin condensation and DNA fragmentation [132]. Members of the Bcl-2 protein family regulate mitochondrial outer membrane permeabilization (MOMP) and are divided into two main groups: anti-apoptotic proteins (e.g., Bcl-2, Bcl-XL, Bcl-W, Bcl-B, A1, and Mcl-1) and pro-apoptotic proteins (including Bax, Bak, and Bok) [133].

Accordingly, various cancer therapies have been developed to target mitochondrial apoptosis, including drugs that affect the inner mitochondrial membrane, inhibit Bcl-2, or activate caspases [131].

Furthermore, apoptosis can be induced by ROS-related stress [126]. When ROS activate p53 and/or c-Jun N-terminal kinase (JNK), they stimulate pro-apoptotic Bcl-2 proteins. This activation inhibits the functions of anti-apoptotic proteins, thereby promoting increased MOMP and cardiolipin oxidation, which, in turn, leads to cytochrome c release [132]. For example, the anticancer drug doxorubicin triggers oxidative stress, thereby activating cell death pathways [134]. Doxorubicin undergoes oxidation to form a semi-quinone, which releases ROS during its reconversion to doxorubicin [135]. Doxorubicin elevates hydrogen peroxide and superoxide levels, depolarizes the mitochondrial membrane, releases cytochrome c, and activates caspase-3. Additionally, it increases BAX protein levels while decreasing levels of the anti-apoptotic BCL-2 protein, further promoting apoptosis [136].

### 4.4. p53 and ROS-Induced Cell Death

p53 is activated in response to cellular stress and functions as a DNA-binding transcription factor that selectively regulates numerous target genes essential for cell cycle arrest, cellular aging, DNA repair, and various cell death pathways [137]. Key p53 targets include Bax, an apoptosis-related gene; p21, a cyclin-dependent kinase (CDK) inhibitor; and 14-3-3σ, a protein that regulates cell cycle progression by inhibiting the nuclear import of the Cyclin B/Cdc2 complex, preventing premature mitosis [138].

Although p53 activation alone is not sufficient to induce ferroptosis, it can promote this form of cell death in the presence of GPX4 inhibitors or elevated ROS levels (Figure 3) [139]. One mechanism through which p53 induces ferroptosis is by suppressing SLC7A11, a key component of the cystine/glutamate antiporter. The inhibition of SLC7A11 reduces cystine uptake, thereby limiting glutathione synthesis and sensitizing cells to ROS-induced ferroptosis [140]. Additionally, p53 activates SAT1 (spermidine/spermine N1-acetyltransferase 1), a rate-limiting enzyme in polyamine degradation, leading to lipid peroxidation and ferroptosis during oxidative stress. This is because SAT1 and polyamine oxidase (PAO) produce H_2_O_2_ during polyamine degradation, enhancing oxidative stress [141]. In cells such as neurons, pancreatic cells, and osteoblasts from Bax/Bak double knockout mice, oxidative stress causes p53 translocation to the mitochondria, where it associates with CypD (cyclophilin D), forming the CypD-p53 complex. This complex promotes the opening of the mitochondrial permeability transition pore (MPTP), leading to necrotic cell death [142]. Evidence further suggests that p53 elevates levels of cathepsin Q, a lysosomal cysteine protease. In the presence of ROS, cathepsin Q may contribute to necrosis [143].

### 4.5. Copper

Copper plays an essential role in various critical biological processes, including energy metabolism, ROS detoxification, iron uptake, and signal transduction in eukaryotes. In the mitochondrial matrix, copper is utilized by respiratory complex IV (cytochrome c oxidase) and the antioxidant enzyme superoxide dismutase 1 [144]. Given copper’s involvement in mitochondrial OXPHOS, both copper deficiency and excess can lead to mitochondrial dysfunction, resulting in increased ROS production and subsequent cell death. An imbalance in copper levels has also been shown to affect cell proliferation and differentiation [144].

Additionally, mitochondrial metabolism renders cancer cells more vulnerable to the effects of a small molecule compound known as elesclomol [145,146,147]. Elesclomol forms a complex with copper in the extracellular space, which is then transported to the mitochondria. Within the mitochondrial matrix, this complex undergoes a reduction in Cu(II) to Cu(I), leading to ROS generation [148]. Once elesclomol dissociates from the complex, it exits the cell and forms a new complex with extracellular copper again, thereby facilitating copper to accumulate in the mitochondria. After releasing Cu within the mitochondria, elesclomol exits the cell and can form a new complex with extracellular copper, thereby promoting copper accumulation within the mitochondria [148]. Because cancer cells often maintain high basal ROS levels, the further oxidative stress induced by elesclomol can push these cells past their threshold, leading to cell death (Figure 3). This selective oxidative stress has little to no effect on normal cells, effectively targeting only cancer cells [149]. Furthermore, many studies report elevated copper levels and increased copper metabolism in various cancers compared to normal tissues, enhancing the cancer cells’ absorption of the elesclomol-Cu(II) complex [150].

FDX1, a mitochondrial reductase involved in synthesizing iron–sulfur (Fe-S) clusters, plays a crucial role in this process. Elesclomol binds directly to reduced FDX1, inhibiting its Fe-S cluster-generating capacity. Consequently, the elesclomol-Cu(II) complex fully oxidizes FDX1, facilitating the reduction in Cu(II) to Cu(I) [151]. Although initially thought to induce cell death through ROS-dependent and apoptotic mechanisms, further research has shown that elesclomol-induced cell death does not involve caspase 3 activation or cleavage, which are typical markers of apoptosis [152]. Instead, cells reliant on cellular respiration exhibit sensitivity to copper ionophore-induced cell death, a mechanism termed cuproptosis. This process selectively targets lipidated proteins within the tricarboxylic acid (TCA) cycle rather than the electron transport chain (ETC) [146].

Copper metabolism, especially through compounds like elesclomol, offers a promising therapeutic approach in cancer treatment. By leveraging the cancer cells’ vulnerabilities to copper ionophore-induced cuproptosis, targeted therapeutic strategies can be designed to selectively induce cell death in tumors with minimal adverse effects on healthy tissues.

### 4.6. Autophagy

Autophagy is a critical cellular process that recycles cellular components and clears damaged organelles under various stress conditions, such as nutrient deprivation, viral infections, genetic mutations, and toxic stress [153,154]. This pathway is primarily mediated by the autophagy-related (ATG) protein 1 (ATG1) and Unc-51-like kinase (ULK) complexes [155]. The Atg1/ULK1 complex is part of the Class III PI3K complex, which includes VPS34, VPS15, Atg6 (BECLIN1), and Atg14. This complex phosphorylates essential proteins involved in autophagosome formation, facilitating their recruitment to generate phosphatidylinositol 3-phosphate (PI(3)P), a crucial phospholipid for isolation membrane formation. Additionally, the Atg8/LC3-phosphatidylethanolamine (PE) system and the Atg12-Atg5 system allow the attachment of Atg8 (LC3) to the autophagosome membrane, a process regulated by various associated proteins [156,157,158,159]. In the final stage, autophagosomes fuse with lysosomes to form autolysosomes, where the degradation of cellular components occurs [160].

In immune cells, such as phagocytes, NADPH oxidase 2 (NOX2) generates ROS that target the autophagy protein LC3 for efficient pathogen clearance within phagosomes [161]. Furthermore, ROS have been shown to facilitate autophagy under nutrient-deficient conditions [162]. Specific reactive oxygen species, such as O_2_•^−^, are produced when cells lack glucose, L-glutamine, pyruvate, or serum, whereas a deprivation of amino acids results in the generation of both O_2_·⁻ and H_2_O_2_ [163]. H_2_O_2_ is a relatively stable ROS that can traverse the mitochondrial membrane, acting as an autophagy inducer [164]. It has been shown that brief exposure to low doses of H_2_O_2_ (15 to 30 min) can transiently induce autophagy [165].

ROS also directly influence autophagy-related proteins. For instance, the cysteine protease Atg4 is particularly susceptible to oxidative modification, where H_2_O_2_ oxidizes and inactivates Atg4. This inactivation prevents LC3 from dissociating from PE on the autophagosome membrane, thereby promoting autophagy [166]. Additionally, ROS activate 5′ AMP-activated protein kinase (AMPK) through upstream activator ataxia-telangiectasia mutated (ATM), which subsequently inhibits mTOR to initiate autophagy [167].

DNA damage caused by oxidative stress also triggers ATM activation, which, along with p53, inhibits mTOR. The nuclear translocation of p53 further promotes the transcription of genes, such as TSC2 and AMPK β subunits, that regulate autophagy [168]. Selective autophagy targets specific cell components in response to various triggers. Types of selective autophagy include aggrephagy, mitophagy, reticulophagy (ER-phagy), xenophagy, and pexophagy, each targeting different substances [169]. Autophagy has complex roles in cancer, functioning as both a tumor suppressor and a promoter [170]. For instance, autophagy can restrict tumor-promoting effects by clearing damaged mitochondria and preventing ROS accumulation during the initial stages of tumorigenesis [171,172]. Conversely, autophagy can also promote tumors; for example, the loss of NRF2 reduces the transcription of ROS-detoxifying genes, and a deficiency in Atg7 prevents the clearance of ROS-producing mitochondria, temporarily increasing ROS and promoting tumor growth [173]. Autophagy cargo receptors (ACRs), such as p62, play diverse roles, including activating tumor-promoting pathways like NF-κB and NRF2 in addition to their cargo functions. Interestingly, adequate p62 levels might also suppress tumor progression under certain conditions [174].

#### 4.6.1. Selective Autophagy

##### Mitophagy

A specialized form of autophagy, termed mitophagy, involves the selective degradation of damaged mitochondria. This process is essential for removing dysfunctional mitochondrial proteins and segments of the mitochondrial network. After clearance, mitochondrial biogenesis restores function by synthesizing and incorporating new proteins and lipids, facilitating the complete turnover of mitochondria [175]. ROS accumulation can lead to oxidative stress and mitochondrial dysfunction, both of which can induce mitophagy. By degrading oxidized cellular components, mitophagy mitigates oxidative damage [176]. Several mitochondrial proteins, referred to as mitochondrial cargo receptors, play critical roles in recognizing damaged mitochondria and directing them to autophagosomes for degradation. These include Bcl-2/adenovirus E1B 19 kDa-interacting protein 3 (BNIP3) [177]; BNIP3-like protein (BNIP3L/NIX) [178]; UN14 domain-containing 1 (FUNDC1) [179]; B-cell lymphoma 2-like protein 13 (Bcl2-L-13) [180]; FK506-binding protein 8 (FKBP8) [181]; prohibitin-2 (PHB-2) [182]; and cardiolipin [173]. These proteins maintain mitochondrial function and structural integrity under stress conditions, aiding in selective mitophagy.

The PINK1–Parkin pathway is essential for mitophagy, tagging damaged mitochondria through ubiquitination and facilitating autophagosome engulfment [183]. The loss of PINK1 and Parkin activity, observed in various cancers, has been associated with cancer progression and metastasis [183,184]. Parkin deficiency can shift metabolism toward glycolysis, reducing mitochondrial respiration and promoting the Warburg effect, a characteristic of many cancers [185]. Parkin also acts as an E3 ubiquitin ligase for hypoxia-inducible factor-1α (HIF-1α), mediating its degradation and thereby impeding metastasis in breast cancer [186]. During mitophagy, PINK1 and Parkin activate TBK1, relocating it from the centrosome to the mitochondria to inhibit cell division [187]. Mitophagy plays a crucial role in maintaining the unique characteristics of cancer stem cells. In liver cancer stem cells, PINK1 phosphorylates p53 within mitochondria, which promotes its degradation through mitophagy. This degradation process is further facilitated by the stem cell factor Nanog [188]. Cancer stem cells, a minor subpopulation of malignant cells, are pivotal in driving tumor initiation and progression. These cells share traits with normal stem cells and express various cell surface markers, including Nanog, which is upregulated in multiple cancers. Nanog commonly interacts with transcription factors like Sox-2 and Oct-4 to regulate pluripotency and self-renewal in embryonic stem cells [189]. The upregulation of Nanog has been associated with tumor development, malignant progression, and poor prognosis across several cancer types [190].

A significant proportion of invasive breast cancers show a loss of BNIP3 expression. Tumors with reduced BNIP3 expression are linked to a higher incidence of lymph node metastasis and increased mitotic activity. In normal breast tissue, BNIP3 is predominantly nuclear, whereas, in breast tumor cells, it shifts to a cytoplasmic localization [191]. BNIP3 is a hypoxia-inducible protein that directs mitochondria to autophagosomes for degradation, thereby preventing the accumulation of dysfunctional mitochondria and excessive ROS production. The loss of BNIP3 compromises mitochondrial clearance, resulting in elevated mitochondrial ROS, which consequently increases the expression of Hif-1α and its target genes [192]. Conversely, high BNIP3 levels in certain cancer types or specific cancer cell populations have been associated with adverse patient outcomes [183].

FUNDC1 phosphorylation enables its binding to LC3, initiating mitophagy [193]. Elevated FUNDC1 expression has been linked to increased proliferation, migration, and invasion in breast cancer cells, which correlates with a poorer prognosis [194]. Studies have shown that hydrogen peroxide exposure in laryngeal cancer cells elevates FUNDC1 levels. This upregulation of FUNDC1 enhances mitophagy, enabling laryngeal cancer cells to better withstand oxidative stress [195].

##### Pexophagy

Peroxisomes are single-membrane-bound organelles present in nearly all eukaryotic cells and play a critical role in various metabolic processes, including the β-oxidation of fatty acids, redox balance, and the synthesis of bile acids and plasmalogens [196]. As both producers and decomposers of ROS, peroxisomes contribute significantly to cellular oxidative balance. During oxidative stress, redox imbalance within peroxisomes can trigger a specific autophagic process known as pexophagy, which selectively degrades peroxisomes to mitigate stress [197]. Catalase, an enzyme housed within peroxisomes, decomposes hydrogen peroxide, a key ROS; the inhibition of catalase raises ROS levels, which can subsequently induce pexophagy [198]. Oxidative stress also activates the ataxia-telangiectasia mutated (ATM) signaling pathway, further promoting pexophagy via a ubiquitin-dependent process involving the PEX5 protein [199]. Most peroxisomal proteins contain a peroxisomal targeting signal 1 (PTS1) recognized by PEX5, which transports PTS1-containing proteins into peroxisomes. After cargo delivery, PEX5 is recycled back to the cytosol for subsequent transport cycles, a process requiring ATP [200]. In approximately 90% of clear cell renal cell carcinoma (ccRCC) cases, the Von Hippel–Lindau (VHL) gene is mutated, affecting multiple cellular pathways [201]. VHL inactivation leads to the stabilization and activation of HIF-2α, a process implicated in cancer development. HIF-2α activation promotes pexophagy, which reduces peroxisome abundance and facilitates the accumulation of fat and sugar in ccRCC cells [202]. To survive in low-oxygen (hypoxic) environments, cancer cells store fatty acids in lipid droplets. Unlike normal cells, which use the protein PNPLA2 (or ATGL) to release fatty acids from these droplets for energy, cancer cells block this release with the help of a protein called hypoxia-inducible gene 2 (HIG2) [203]. HIG2, overexpressed in various cancers—including ccRCC, colorectal adenocarcinoma, bladder urothelial carcinoma, lung squamous cell carcinoma, and uterine corpus endometrial carcinoma—facilitates lipid droplet accumulation, protecting cells from lipotoxicity and enabling growth in low-oxygen environments [204]. In a ccRCC mouse xenograft model, HIF-2α overexpression was associated with increased tumor growth, whereas inhibiting HIF-2α through RNA interference reduced tumor progression, highlighting HIF-2α as a potential therapeutic target [205].

##### Aggrephagy

Oxidative stress has been shown to both initiate and sustain protein aggregation [206]. All living organisms rely on mechanisms to selectively degrade misfolded and aggregated proteins. Without these processes, such proteins accumulate, potentially leading to neurodegenerative diseases like Alzheimer’s disease (AD) and Parkinson’s disease (PD) and facilitating cancer progression [207,208]. Key proteins involved in aggregate clearance include p62 and NBR1, which recognize aggregated proteins and aid in forming autophagosomes via interactions with TAX1BP1. These autophagosomes then fuse with lysosomes, enabling the degradation of protein aggregates [209]. The overexpression of p62 has been observed in various cancers and is associated with poor prognosis [210]. While the role of NBR1 in cancer remains uncertain, decreased NBR1 mRNA levels have been correlated with unfavorable outcomes in ccRCC [211,212]. However, other studies suggest that NBR1 might have tumor-promoting properties [213]. NBR1 also participates in the regulation of cancer cell migration, a process tightly controlled by focal adhesions (FAs), which are protein complexes that anchor tumor cells to the extracellular matrix (ECM) via integrins [213]. The assembly and disassembly of FAs are essential for controlling migration efficiency in tumor cells, thereby enhancing tumorigenic properties [214].

In HRAS-transformed MCF10A breast cancer cells, which model early tumor progression, NBR1 interacts with ubiquitinated FA proteins, facilitating their degradation through autophagy. Decreasing NBR1 levels impairs FA turnover, reducing breast cancer cell migration [215]. Tumor cells often evade CD8+ T cells due to mutations or the decreased expression of major histocompatibility complex class I (MHC-I) molecules [211]. NBR1 has been shown to interact with ubiquitinated MHC-I, indicating that MHC-I surface distribution is regulated by NBR1-selective autophagy. This finding underscores NBR1’s importance in tumor immune evasion [216]. The tumor microenvironment, which includes malignant cells, immune cells, stromal cells, and soluble factors, plays a significant role in acute myeloid leukemia (AML) pathogenesis by promoting tumor progression and immune escape [217]. In AML, distinct clusters related to aggrephagy—a selective autophagy targeting protein aggregates—were identified in T cells, natural killer (NK) cells, and myeloid cells. Specific gene expression patterns related to aggrephagy in these clusters were linked to patient survival outcomes. Interestingly, higher numbers of certain aggrephagy-related clusters were associated with improved responses to immunotherapy [218].

##### Reticulophagy

ER is a multifunctional organelle that plays a critical role in cellular processes. In cancer cells, protein synthesis is often upregulated, leading to an accumulation of misfolded proteins and resulting in ER stress, which can ultimately trigger cell death [116]. ROS have also been shown to induce ER stress [117]. To alleviate ER stress, cells may initiate reticulophagy—a specialized form of autophagy that selectively targets and degrades portions of the ER under stress conditions. Several receptors facilitate reticulophagy in mammalian cells, including Family with Sequence Similarity 134 Member B (FAM134B), Alastin-3 (ATL3), Reticulons (RTN1-4), SEC62, CCPG1, and TEX264 [219,220]. FAM134B, in particular, is frequently overexpressed in esophageal squamous cell carcinoma (ESCC) and has been shown to drive cellular transformation [221]. However, in colorectal cancer, a lower FAM134B expression has been associated with advanced disease stages [222]. Studies have demonstrated that the overexpression of FAM134B can effectively reduce intracellular ROS accumulation, apoptosis, and senescence in response to advanced glycation end-products (AGEs), which otherwise elevate cell death, senescence, and ROS levels [223]. Consequently, increasing ER stress in cancer cells has emerged as a potential therapeutic strategy for tumor management [224]. Nevertheless, since ER stress can also induce reticulophagy—a survival pathway for cancer cells—it may be beneficial to inhibit this process. For example, berberine has been shown to effectively inhibit reticulophagy, thereby potentially preventing cancer cells from utilizing this stress-relief mechanism for survival [225].

##### Xenophagy

Xenophagy is an innate cellular defense mechanism that targets various intracellular pathogens by capturing them for lysosome-mediated degradation. This process also enhances adaptive immunity by increasing antigen presentation [226]. Pathogens, such as bacteria, often enter host cells through phagocytosis or endocytosis, which typically leads to degradation via fusion with endosomes and lysosomes. However, some bacteria have evolved to evade this pathway by blocking lysosome fusion [226].

In the cytosol, pathogens targeted for xenophagy are ubiquitinated, facilitating their recognition by autophagosomes. These autophagosomes then fuse with lysosomes, leading to bacterial degradation [227]. Xenophagy also promotes immune responses by delivering viral components to endosomes containing Toll-like receptors, thereby enhancing the recognition of viral particles [228]. Certain bacteria, including *Helicobacter pylori* (*H. pylori*), are associated with cancer development. *H. pylori* infection is strongly linked to gastric cancer and involves two primary proteins, VacA and CagA. VacA has been shown to trigger xenophagy; however, chronic VacA exposure can disrupt xenophagy, resulting in the accumulation of ROS and toxic substances. This accumulation is linked to inflammation and cancer progression. Similarly, CagA, which is typically degraded through xenophagy, can accumulate in cancer stem cells where xenophagy is impaired, further promoting tumor development [229]. Conversely, some bacteria may induce xenophagy as a means of promoting cancer cell death [229]. For example, *Salmonella typhimurium* activates xenophagy in melanoma cells. When *S. typhimurium* accumulates at tumor sites, cancer cells initiate xenophagy to eliminate the bacteria. This process is regulated by the AKT-mTOR pathway, which redirects cellular resources from proliferation to xenophagy activation, thereby inhibiting tumor growth [230].

## 5. Cancer and ROS

Cancer cells often exhibit higher levels of ROS compared to normal cells [231]. While elevated ROS levels can inflict cellular damage, potentially compromising cell survival, they can also drive cancer development by inducing DNA damage and promoting genomic instability [232]. Increased ROS levels have been observed across various cancer types and are associated with several key effects, including the activation of cancer-related signaling pathways, enhanced cell survival and proliferation, and heightened genetic instability [233].

### 5.1. Cancer Cell Survival, Growth

The transcription factor HIF-1α is crucial for enhancing glycolysis and promoting tumor growth in hypoxic environments [234]. Under normal conditions, prolyl hydroxylase (PHD) targets HIF-1α for degradation; however, reduced PHD activity in tumors stabilizes HIF-1α, resulting in its accumulation and the subsequent promotion of glycolysis to support tumor growth [235]. Additionally, HIF-2α induces the expression of pro-survival factors such as Nanog, Oct4, and c-MYC, further supporting tumor growth and survival under hypoxic conditions [236].

ROS also influences various signaling pathways that regulate cell growth by reversibly oxidizing proteins. This modulation affects protein tyrosine phosphatases, protein tyrosine kinases, receptor tyrosine kinases, and transcription factors. Additionally, ROS has been shown to activate key signaling pathways, including the MAPK/ERK cascade, PI3K/Akt signaling, and NF-κB pathways [237].

Although cancer cells generally display increased glucose uptake and glycolytic metabolism, certain areas within tumors experience glucose depletion due to rapid consumption and limited nutrient availability in the tumor microenvironment [238]. Glucose deprivation reduces ATP production and increases ROS levels. In response, AMPK, a critical cellular energy sensor, becomes activated to support cell survival under low-energy conditions [239].

The chronic secretion of elevated ROS levels, particularly in the context of persistent inflammation, can attract more immune cells to the tumor microenvironment, intensifying disease progression and potentially leading to a precancerous state [240]. Antioxidants such as GSH and thioredoxin influence cancer progression by reducing ROS levels and preventing cell death [241]. Moreover, mutations in NRF2, which commonly occur in squamous cell carcinomas, impair its interaction with Keap1, a protein that mediates NRF2 degradation. This leads to enhanced NRF2 activity, enabling cancer cells to better withstand oxidative stress [242]. RAC1, a GTPase that activates NOX at the cell membrane, is upregulated when the APC tumor suppressor gene is lost. The active form, RAC1B, is associated with cancers such as melanoma and lung cancer, where it promotes oncogenesis by increasing mitochondrial ROS (mtROS), contributing to oxidative stress, and facilitating tumor progression by enhancing cellular instability and survival pathways [20].

### 5.2. ROS in the Tumor Microenvironment (TME)

In immune cells, ROS play a vital role in processes such as phagocytosis, antigen processing and presentation, cell lysis, and phenotypic differentiation. However, ROS can also suppress the function of T cells and natural killer (NK) cells, contributing to the immunosuppressive environment commonly found in solid tumors. This immunosuppressive effect is especially prominent in the tumor microenvironment (TME), where it aids tumor growth by dampening immune responses against tumor cells [243]. The TME provides oxygen, nutrients, growth factors, and cytokines, all of which support tumor growth and simultaneously work to inhibit immune responses [244]. Antitumor immunity is initiated when dendritic cells capture tumor-associated antigens and present them to CD8+ T cells, which then become activated, migrate to the tumor site, and specifically target and destroy tumor cells [245]. ROS play a role in antigen processing and presentation by dendritic cells; NOX2-generated ROS aid in raising the pH within phagosomes, which is essential for antigen degradation and subsequent T cell presentation [246,247]. However, excessive ROS levels can trigger activation-induced cell death (AICD) in T cells, a regulatory process that causes T cell depletion upon antigen-driven activation, allowing tumors to evade immune detection [248]. ROS also promote the differentiation of regulatory T cells (Tregs), which act to prevent autoimmunity by suppressing T cell responses, thereby reducing tumor immunity [249]. Furthermore, ROS released by tumor cells influence macrophages to assume an M2 phenotype via PI3K signaling; M2 macrophages secrete growth factors and cytokines that support tumor cell proliferation [250]. Additionally, the stabilization of HIF-1α within tumors promotes the transcription of PD-L1, a common immune evasion strategy utilized by tumors [251]. Elevated ROS levels can also reduce CD16ζ expression in NK cells, which is crucial for their cytotoxic response and ability to recognize and destroy target cells. Therefore, high ROS levels may impair NK cell function by diminishing CD16ζ expression, limiting their effectiveness in targeting tumors [252].

### 5.3. ROS in EMT

Epithelial–mesenchymal transition (EMT) describes a morphological change from epithelial to mesenchymal cell types, enhancing cell motility and invasiveness, thereby promoting tumor progression and drug resistance [98]. Beyond facilitating metastasis, EMT also induces cancer stem cell (CSC) characteristics, marked by the expression of stem cell markers, sphere-forming capability in vitro, tumorigenic potential, and resistance to treatment [253]. HIF-1α has been shown to play a critical role in driving EMT in both kidney cells and hepatocytes [254,255]. Mitochondrial ROS have been identified as a significant factor in hypoxia-induced EMT in alveolar epithelial cells, with further evidence the supporting role of ROS in promoting EMT in various cell types [256,257]. Increased ROS levels have been associated with the reduction in E-cadherin, an epithelial marker, through an ERK-dependent pathway [258]. Additionally, ROS stimulates the expression of transforming growth factor-β (TGF-β), which activates the SMAD protein complex. This complex, in turn, induces the expression of mesenchymal genes and transcription factors, such as Snail, Twist, Slug, and ZEB1, which drive the EMT process. Consequently, there is an upregulation of mesenchymal markers like vimentin and fibronectin and a concurrent downregulation of E-cadherin expression [2].

### 5.4. Cancer Treatment Using ROS

Therapeutic strategies targeting the high-ROS environment of cancer cells include the following: In one study, researchers designed a ROS-sensitive polymer to create nanoparticles (NP@ESCu) containing elesclomol (ES) and copper. These nanoparticles were administered to cancers with high ROS concentrations, inducing cuproptosis—a form of programmed cell death that relies on copper [259]. Radiation therapy, which generates ROS in cancer cells to induce DNA damage, can also harm normal tissues. To address this limitation, nanoparticles with a core–shell structure made from a metal–semiconductor (Au@AgBiS_2_) were developed to induce cell death processes more selectively within tumors [260]. In another study, melatonin, a hormone that regulates sleep, was found to promote mitochondrial reverse electron transport (RET), increase ROS production, and exert tumor-suppressing effects through apoptosis induction [261]. This suggests the potential for developing cancer treatments with reduced side effects using melatonin. The antidiabetic drug metformin inhibited the proliferation of breast cancer cells by increasing intracellular Fe^2^⁺ and lipid ROS levels, inducing ferroptosis through the destabilization of SLC7A11—a regulator of ferroptosis [262]. Artesunate, an antimalarial drug and promising candidate for colon cancer therapy, promotes autophagy by stimulating excessive ROS production [263]. These studies illustrate the potential for drug repurposing, which could contribute to developing cancer treatments with fewer side effects.

Conversely, targeting the antioxidant system in cancer cells may offer another therapeutic approach. The thioredoxin (Trx) antioxidant system, a major redox regulatory mechanism, is overexpressed in various tumors, and its inhibitors include 1-methylpropyl 2-imidazolyl disulfide (IV-2, also known as PX-12) and suberoylanilide hydroxamic acid (SAHA) [264]. Given the complexity of endogenous antioxidant mechanisms in cancer cells, researchers initially believed that simply inhibiting individual antioxidant pathways might have limited efficacy. Thus, a versatile nanoparticle-based drug called PZB NP was developed, containing L-buthionine sulfoximine (BSO), a glutathione inhibitor, and zinc protoporphyrin (II) (ZnPP), an inhibitor of heme oxygenase-1 (HO-1). These components synergistically inhibit the innate antioxidant defenses of cancer cells, leading to cell death [265].

This multifaceted approach highlights both direct ROS-inducing agents and antioxidant system inhibitors as potential strategies for effective cancer therapies.

## 6. Conclusions and Further Direction

ROS are highly reactive molecules that play a complex, dual role in cancer biology [2,13]. On one hand, ROS function as signaling molecules that contribute to genomic instability, activate oncogenic pathways, and promote cancer cell proliferation, migration, invasion, angiogenesis, and drug resistance. Conversely, ROS can also trigger cell death pathways, such as apoptosis and autophagy, presenting a potential therapeutic avenue in cancer treatment [5]. ROS-induced DNA damage can lead to mutations and chromosomal abnormalities, which drive tumor formation [38]. Furthermore, ROS contribute to the activation of survival pathways, including NF-κB, PI3K/Akt, and NRF2, which enhance tumor cell survival, proliferation, and resistance to apoptosis [21]. Under specific conditions, ROS can also initiate programmed cell death pathways, such as apoptosis, autophagy, ferroptosis, and ER stress-induced apoptosis, which can suppress tumorigenesis [127,161,162,164].

By elucidating the conditions under which ROS either promote or inhibit cancer progression, researchers can develop targeted therapies that either reduce ROS levels to prevent cancer development or elevate ROS to induce cancer cell death. Therapeutic strategies harnessing ROS include ROS inducers, targeting antioxidant systems to sensitize cancer cells to ROS-induced damage and inhibiting survival pathways, such as NF-κB and PI3K/Akt, in synergy with ROS. These approaches demonstrate the potential for ROS-based cancer therapies. However, a balanced approach is essential to harness the dual roles of ROS effectively within cancer cells. In the context of personalized medicine, it is also essential to tailor ROS-modulating strategies based on the unique redox profiles of individual tumors. The development of reliable biomarkers to monitor ROS levels in real time could significantly enhance therapeutic precision.

## Data Availability

All the references are cited in the manuscript; however, we apologize for the omission of any primary citations.
